# In-column ATR-FTIR spectroscopy to monitor affinity chromatography purification of monoclonal antibodies

**DOI:** 10.1038/srep30526

**Published:** 2016-07-29

**Authors:** Maxime Boulet-Audet, Sergei G. Kazarian, Bernadette Byrne

**Affiliations:** 1 Department of Chemical Engineering, Imperial College London, South Kensington Campus, London, SW7 2AZ, UK; 2 Department of Life Sciences, Imperial College London, South Kensington Campus, London, SW7 2AZ, UK

## Abstract

In recent years many monoclonal antibodies (mAb) have entered the biotherapeutics market, offering new treatments for chronic and life-threatening diseases. Protein A resin captures monoclonal antibody (mAb) effectively, but the binding capacity decays over repeated purification cycles. On an industrial scale, replacing fouled Protein A affinity chromatography resin accounts for a large proportion of the raw material cost. Cleaning-in-place (CIP) procedures were developed to extend Protein A resin lifespan, but chromatograms cannot reliably quantify any remaining contaminants over repeated cycles. To study resin fouling *in situ*, we coupled affinity chromatography and Fourier transform infrared (FTIR) spectroscopy for the first time, by embedding an attenuated total reflection (ATR) sensor inside a micro-scale column while measuring the UV 280 nm and conductivity. Our approach quantified the in-column protein concentration in the resin bed and determined protein conformation. Our results show that Protein A ligand leached during CIP. We also found that host cell proteins bound to the Protein A resin even more strongly than mAbs and that typical CIP conditions do not remove all fouling contaminants. The insights derived from in-column ATR-FTIR spectroscopic monitoring could contribute to mAb purification quality assurance as well as guide the development of more effective CIP conditions to optimise resin lifespan.

Monoclonal antibodies (mAbs) have emerged as one of the most important classes of biotherapeutics. The high specificity of mAbs means that they bind to target molecules very effectively, reducing the risk of therapeutic side effects^1^. However, the cost of production of biotherapeutic antibodies is considerably higher than that of small molecule drug manufacture due largely to stringent purity requirements imposed by regulatory bodies^2^. For instance, the World Health Organisation, recommends host cell protein (HCP) and DNA limits of 100 ppm and 10 pg per dose respectively^3^,^4^,^5^,^6^. To ensure that the appropriate purity has been achieved following purification, qPCR can quantify trace amounts of host cell DNA^7^, while enzyme-linked immunosorbent assay (ELISA) are usually used to measure levels of HCP and protein A ligand leaching as a result of enzymatic cleavage^8^,^9^.

To achieve high protein purity, the culture fluid first undergoes depth filtration before successive preparative chromatography steps^10^,^11^. The first step affinity chromatography can clear over 98% of HCP and inactive protein fragments in a single step with a ligand designed to bind only the appropriately folded full-length mAb product^2^,^12^,^13^, prior to anion and cation exchange chromatography steps which remove most remaining impurities^10^,^14^,^15^,^16^.

For mAb capture, Protein A cross-linked to agarose is most commonly used as the matrix^17^,^18^,^19^,^20^, but harder silica matrices have also been developed^21^. The affinity chromatography step is usually regarded as the bottleneck of the mAb purification process due to relatively low throughput. Ingenious semi-continuous processes have been developed to overcome this limitation^22^, but most industrial processes operate in batch mode. With a price of around 2000 $/kg^2^,^23^, Protein A affinity resin costs over 30 times more than other types of resin^24^. Unfortunately, cheaper alternatives using *de novo* synthetic ligands do not offer the same specificity and level of HCP clearance^10^,^12^,^25^.

However, the binding capacity of affinity resin decays over repeated purification cycles^6^,^20^,^26^,^27^. Depending on the required purity of the sample, the resin needs to be replaced after 80 to 200 cycles^20^,^26^. Binding capacity decay makes affinity resin the most expensive consumable for mAb production, representing over 50% of the raw material cost^2^. Thus, pharmaceutical companies have a strong incentive to extend resin lifetime through improvement of purification strategies^22^,^28^.

The causes of binding capacity decay remain elusive despite several previous studies. Fouling by irreversible protein binding may be responsible for limiting access to the protein ligand, reducing binding capacity. Culture fluid containing mAb product appears to cause more fouling than null-cell culture fluid^29^. Protein fouling can occur during mAb capture or following low pH elution. The low pH employed during elution promotes aggregation of mAbs^30^ which could then become trapped in the resin pores^10^,^14^,^26^,^29^. Moreover, hydrophobic HCPs such as histone^8^ and antibody fragments can bind to the mAb product during capture to form mixed protein aggregates^29^. Such aggregates are detectable using a range of techniques such as CD, DSC, micro-rheology, Raman, analytical ultra-centrifugation, and light scattering^4^.

To clear non-eluting proteins from the resin, a wide range of cleaning-in-place (CIP) protocols were developed^18^,^28^,^31^. CIP typically involves flowing diluted sodium hydroxide through the column between purification cycles to hydrolyse deposits while sanitizing the resin^28^,^31^,^32^. A reducing solution followed by a chaotropic solution also proved an effective CIP strategy^28^,^33^. This alkaline treatment extends resin lifespan, but it also appears to decrease the binding capacity^26^ due to either Protein A leaching^4^,^34^,^35^ or ligand denaturation^36^. Under alkaline conditions, asparagine and glutamine residues in Protein A are susceptible to deamidation which also decreases binding capacity^37^,^38^. Substitution of these residues resulted in a mutant Protein A with enhanced alkaline resistance^17^. Branded MabSelect SuRe, this more resistant affinity resin rapidly became the market leader^20^.

However, our previous work suggested that sodium hydroxide affects the protein conformation of the ligand, even in the MabSelect SuRe resin^36^. Resin lifespan depends highly on operating conditions, sample preparation, and sample origin^39^. These variables usually leave room for further CIP protocol optimization^26^,^28^.

Based on post-column UV absorption, high throughput static binding capacity assays measure unbound mAbs after elution, enabling the study of many different experimental conditions^28^,^36^. Dynamic binding capacity (DBC), more representative of the purification process, is also widely employed to assess resin lifespan^19^,^26^,^27^. DBC describes the amount of sample that will bind to a resin packed in a column under defined conditions. Calculating the height equivalent to theoretical plate (HETP) quantifies the column’s separation potential^26^,^41^. The shape of the elution peak indicates the lifespan decay^4^. Multivariate analysis of several chromatographic variables can enhance the precision of lifespan estimation^40^.

The analysis of cleaning eluents by surface-enhanced laser desorption/ionization time-of-flight mass spectrometry (SELDI-TOF-MS) and 2D-PAGE can provide details of the chemical profile of the fouling contaminants^28^,^41^,^42^. Unfortunately, mobile phase analysis does not reveal bound fouling contaminants while preserving the resin intact. Transmission and scanning electron microscopy of fouled resin beads clearly showed irreversible containment accumulation^6^,^14^,^31^,^43^. Although these studies were very informative, they were performed on dried resin beads, bearing little resemblance to the hydrated gel^44^. Direct measurement of hydrated resin is required to gain more detailed insights into fouling.

Direct in-column analyses are more representative of the chromatographic media. Confocal Laser Scanning Microscopy (CLSM) enabled direct visualization of protein binding *in situ*^6^,^14^,^29^,^45^. Such an approach combined with labelled proteins offers enhanced sensitivity for fluorescent light imaging^46^. Magnetic resonance imaging^47^ and X-ray computed tomography^48^ both offer in-column non-invasive probing of packed beads but the signal measured lacks specificity to potential fouling constituents and resin.

To investigate resin fouling *in situ*, we chose Fourier transform infrared (FTIR) spectroscopy as a detection method. FTIR spectroscopy is a non-destructive and label-free method capable of measuring gases, liquids or solids. In addition, FTIR spectroscopy allows quantification of solute^49^,^50^. The fast response of infrared detectors can monitor rapid dynamic processes such as samples flowing from a chromatographic column^43^,^51^. Organic molecules absorb mid-infrared light of specific frequencies, resulting in highly individual infrared spectra giving each species a distinct chemical footprint.

The fact that positions of the amide bands in infrared spectra are dependent on the protein secondary structure is particularly relevant for protein characterisation^49^,^52^,^53^,^54^,^56^,^57^,^58^,^59^,^60^. For instance, this detection method has been successfully applied to protein aggregate analysis^49^,^61^,^62^. FTIR spectroscopy thus enables the quantification of both antibody load and HCP impurity concentration in cell culture fluid with a detection limit around 0.7 mg/mL^60^,^63^ and can discriminate between the different constituents of the affinity chromatographic bed^36^. Agarose beads were previously studied *in situ* by infrared spectroscopy in transmission mode. Since water absorbs strongly in the mid-IR range, the transmission cell path length cannot be thicker than several micrometers, limiting the analysis to a single layer of squashed beads of small diameter^64^.

Attenuated total reflection (ATR) overcomes the optical path length limitation by probing only a layer of a few micrometers adjacent to the surface of the ATR crystal^36^,^49^,^56^,^64^, to study protein adsorption^36^,^49^,^57^,^58^,^59^,^65^. As contaminants concentrate mainly on the outer layer of beads^29^, ATR should be particularly sensitive to irreversibly adsorbed protein. Previously, in-column ATR-FTIR spectroscopic detection was only reported for chiral liquid chromatography on mesoporous silica beads smaller than 20 μm^43^. However, recent work from our group demonstrates ATR-FTIR spectroscopy to be an effective means of measuring unaltered hydrated affinity resin beads of diameter ranging from 50 to 150 μm by applying a small controlled load on the resin bed^36^. Building on our earlier studies, here we embedded an ATR-FTIR spectroscopic detector within an affinity liquid chromatography column for the first time and exploited the advantages of in-column ATR-FTIR spectroscopy to study the fouling of Protein A affinity resin *in situ*. Our microchip-based approach measured the purification process of a common mAb using the MabSelect SuRe standard resin widely used in industry^20^. The in-column detection revealed the nature of fouling contaminant build-up during mAb capture and elution. Our powerful approach also revealed the effectiveness of cleaning reagents offering a powerful tool to optimise CIP strategies.

## Results

### ATR-FTIR spectroscopy of affinity resin beads and mAb culture fluid

Probing resin by mid-infrared spectroscopy in transmission mode poses a major challenge, as a single layer of hydrated beads extinguishes most of the light, saturating the absorption. The evanescent wave produced by attenuated total reflection (ATR) allows probing of the surface layer of resin beads (50–150 μm diameter), but relies on an intimate contact between the internal reflection element and the sample. Since agarose beads are convex microspheres, only a small fraction of each bead would interact with the evanescent wave^66^. Hence, resin beads simply sedimented by gravity alone do not appreciably absorb in ATR mode^36^.

**Figure 1:**
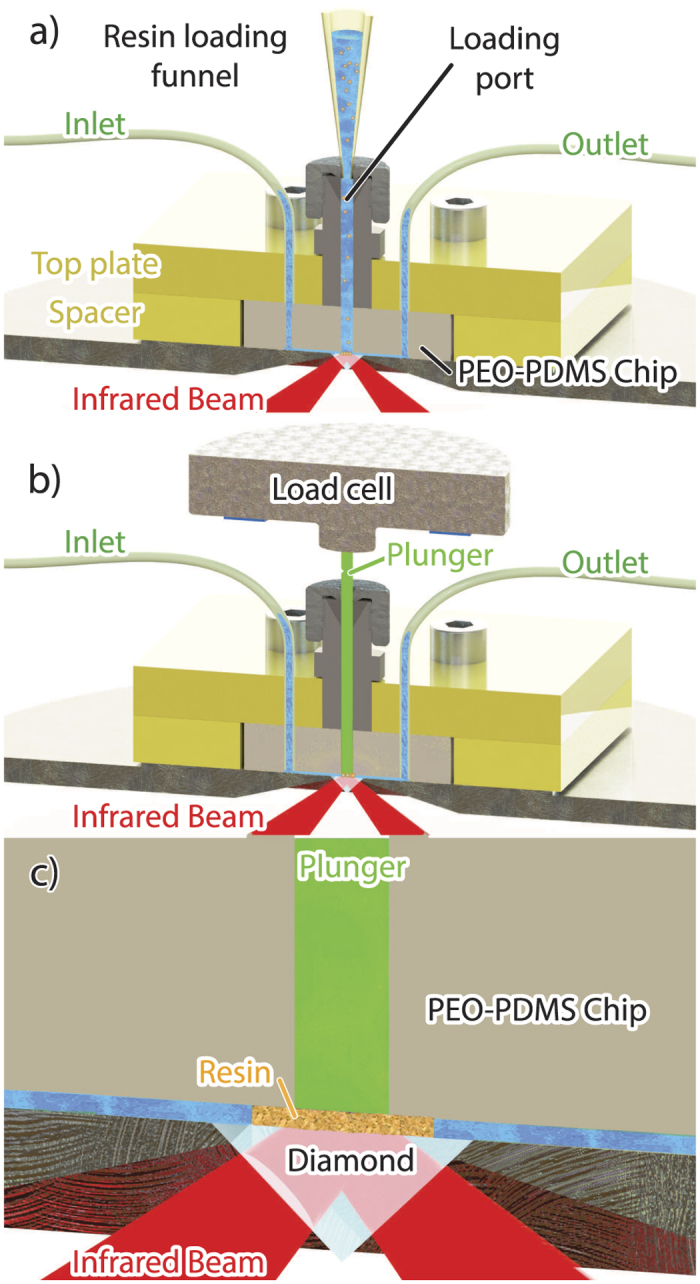
(**a**) Schematic of the experimental set-up for in-column ATR-FTIR spectroscopy with fitted funnel for resin loading. The poly-dimethylsiloxane (PDMS) cell was clamped to the ATR accessory using an acrylic top plate (yellow) connected to both the inlet and outlet. (**b**) Schematic of the experimental setup with the polyether ether ketone (PEEK) plunger (green) securing the resin bed while buffer flows from the inlet to the outlet. The force applied was measured by a load cell mounted on top of the plunger. (**c**) Close-up view of the probed volume inside the microchip.

As in a standard gel chromatography column^18^, we loaded resin beads in a column by sedimentation (Fig. 1a) before compacting the chromatographic bed using a plunger to fill the void between the beads (Fig. 1b). The packed resin formed an intimate contact with the internal reflection element allowing measurable infrared light absorption while preserving the internal pores for the mobile phase to flow through (Fig. 1b). This in-column ATR-FTIR spectroscopic approach allows measurement of the stationary phase resin bed as well as the applied mobile phase and culture fluid.

**Figure 2:**
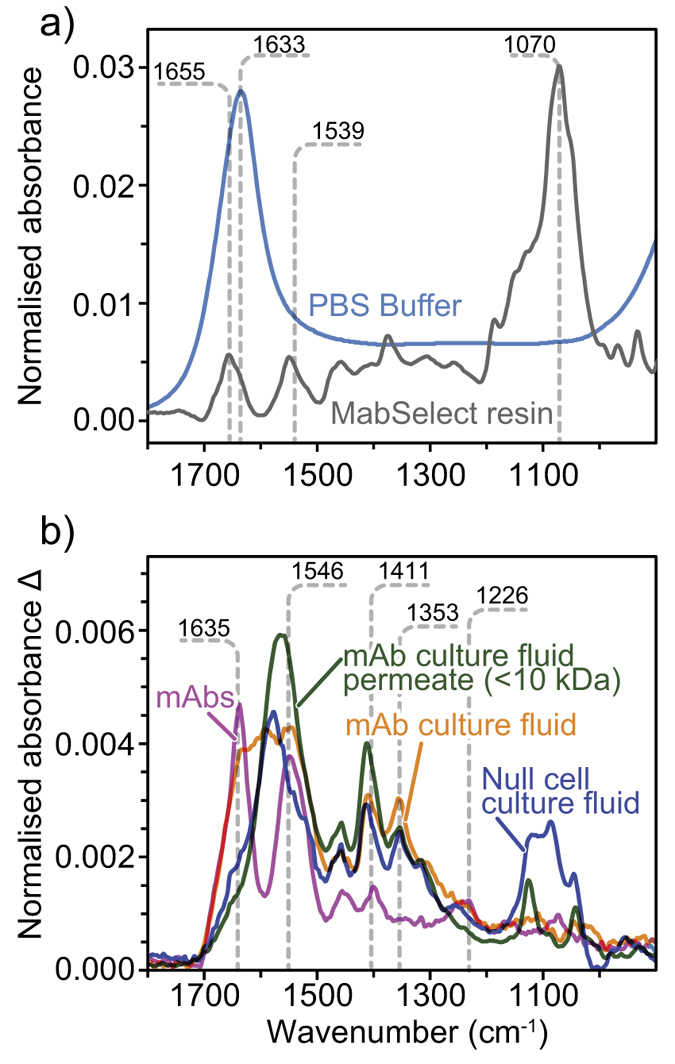
(**a**) Normalized ATR-FTIR spectra of pH 7.4 buffer (blue) and packed MabSelect Sure resin (grey). (**b**) ATR-FTIR difference spectra of IgG4c (orange) culture fluid compared to purified mAbs (pink), mAb culture fluid permeate (<10 kDa) (green) and null cell culture fluid (blue).

Figure 2a compares the ATR-FTIR spectra of 50 mM phosphate buffer and MabSelect affinity resin packed in the microchip. Because of the buffer concentration, phosphate bands at around 1100 cm^−1^ are much weaker than the water bending mode at 1633 cm^−1^. To subtract the contribution of water, resin spectra were calculated against the background of a cell filled with buffer. The spectrum of MabSelect Protein A resin showed agarose peaks at low frequencies, including the 1070 cm^−1^ sugar C-O stretching mode^36^. Since the absorbance measured depends on the amount of resin probed by the evanescent wave^36^,^65^, ATR-FTIR spectra are affected by the contact between resin beads and the internal reflection element. ATR-FTIR spectral bands proved useful to ensure consistent packing between samples. For each measurement, the signal of the agarose peak at 1070 cm^−1^ was adjusted to 30 mA, but could reach 0.2 A upon compression. Under 0.4 ml/min flow, the back pressure increased only from 0.07 MPa to 0.15 MPa after packing the resin, indicating that pores were still accessible to the buffer. The amide I and II bands, around 1652 and 1539 cm^−1^ respectively, reveal the presence of the bioengineered Protein A derivative affinity ligand. The reported protein ligand density was estimated as 5.6 mg/mL^17^. The negative absorbance around 1700 cm^−1^ originates from the displacement of water by agarose.

To assign the numerous overlapping bands of the raw culture fluid with 0.75 mg/mL mAbs (orange on Fig. 2b), we separated the different constituents. A purified mAbs solution (magenta) shows clear Protein Amide I, II and III bands at 1635, 1546 and 1226 cm^−1^ respectively^36^,^49^,^50^. Solutions of known mAb concentration were employed to quantify the protein concentration in the probed volume using a partial least square (PLS) method on the derivative spectra. Several side chain vibrational modes appear in the 1300 to 1500 cm^−1^ region including the 1411 cm^−1^ δCH_2_ and δ_s_CH_3_ 1353 cm^−1^ alanine or glycine component^67^. Using a <10 kDa cut off, the culture fluid permeate (green) does not show dominant amide bands, but a stronger peak at 1585 cm^−1^ assigned to δNH_2_ of glycine from the media feed^67^. The spectrum of the null-cell culture fluid appears similar to the culture fluid permeate, indicating that both contain low protein and high amino acid concentrations. The amino acid peak at 1585 cm^−1^ overlaps with the amide band region, interfering with the protein concentration quantification.

### In-column ATR-FTIR spectroscopy of CIP

Cleaning-in-place (CIP) is commonly employed to regenerate affinity columns between purification cycles and prevent fouling contaminant build-up^25^,^26^,^32^,^35^,^36^,^40^. However, CIP alone appears to reduce binding capacity by either causing cleavage or denaturation of the Protein A ligand^4^,^34^,^35^,^36^. Instead of measuring Protein A leaching, our in-column ATR-FTIR spectroscopic approach aimed at quantifying ligand density underflow *in situ* during CIP while measuring the absorbance at 280 nm. Our spectra-based PLS method quantified the in-column protein density of MabSelect resin packed in the microchip device with a detection limit of ~0.1 mg/mL, less than 1 μg of protein in a 10 μL resin probed. Collecting a background of MabSelect resin under buffer flow revealed the effect of CIP over repeated cycles.

**Figure 3:**
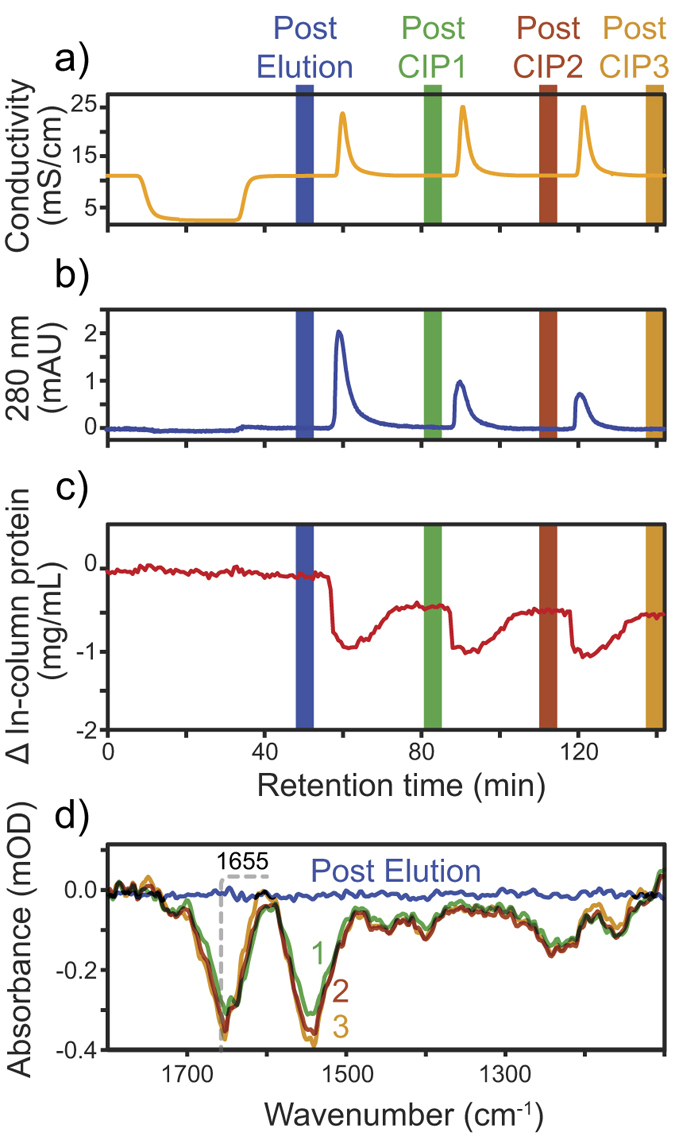
(**a**) Conductivity and (**b**) 280 nm absorbance of the mobile phase during 400 mM NaOH CIP cycles. (**c**) In-column protein concentration difference (red) calculated from ATR-FTIR spectra using PLS regression. (**d**) ATR-FTIR spectra after pH 3.0 elution and subsequent 400 mM NaOH CIP.

To compare with a typical purification protocol^28^, Protein A resin was first exposed to low pH buffer. Figure 3a shows the conductivity (orange) dropping when 10 mL of pH 3.0 buffer was added in the absence of NaCl. Reintroducing binding buffer at pH 7.4 and 100 mM NaCl returned the conductivity to ~10 mS/cm. The resin was then cleaned with three CIP cycles using 200 μL of 400 mM NaOH in the absence of NaCl, giving three conductivity peaks of ~22 mS/cm.

Figure 3b shows the UV absorbance at 280 nm (blue) with only weak peaks detectable during CIP. No peaks were observed during the pH 3.0 elution as the resin appeared stable under such conditions. During the first CIP cycle however, the absorption reached 2 mAU while subsequent cycles resulted in peaks weaker than 1 mA. UV absorbance measurements imply that the first exposure to 400 mM NaOH leached more protein than the following CIP cycles. Because the CIP buffer absorbed UV slightly more than the binding buffer, integrating the peak does not directly relate to the amount of ligand leached. Quantifying the amount of leaching protein during CIP can be performed using ELISA^9^, but the remaining ligand density concentration is challenging to quantify without measuring the resin *in situ*.

While UV 280 nm shows what flows out of the column, the ATR-FTIR spectra reveal the cumulative effect of CIP on the column. Figure 3c (red) shows the in-column ligand density remaining constant throughout the elution before decreasing by around 0.5 mg/mL after the first 400 NaOH CIP. In agreement with the UV 280 nm, subsequent CIP decreased the protein density much less. Assuming a 5.6 mg/mL ligand density^17^, ATR-FTIR spectra indicate that more than 90% of the ligand remained after three cycles of 400 mM NaOH, equivalent to a 1.5-minute exposure. This result confirms the resistance of MabSelect to harsh CIP conditions.

Figure 3d presents the difference spectra collected after each CIP cycle with negative amide bands from protein leaching. The peak at 1655 cm^−1^ also corresponds to the Protein A ligand with predominantly helical structures. The lack of difference in the C-O stretching region around 1050 cm^−1^ suggests that the agarose matrix remains unaffected by the CIP cycles with 400 mM NaOH.

400 mM NaOH lies in the upper range of alkaline conditions usually employed for CIP^28^. To represent the conditions typically used for CIP, we also tested a range of milder CIP conditions; both lower concentrations of NaOH or a reducing solution of thioglycerol followed by a chaotropic solution of guanidine hydrochloride, an alternative effective CIP strategy^33^.

**Figure 4:**
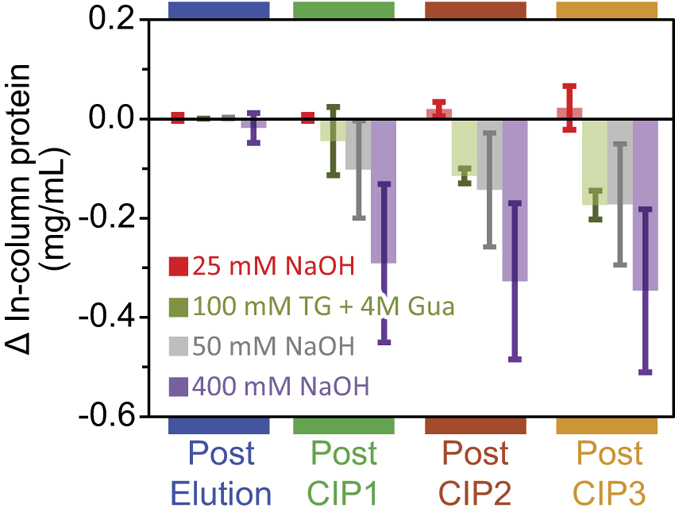
In-column protein concentration difference calculated from ATR-FTIR-based PLS regression (red) after elution and CIP for different conditions. The error bars represent the 95% confidence interval for n = 3.

Figure 4 shows the in-column protein concentration difference after three CIP cycles under different buffer conditions. 400 mM NaOH leached the most Protein A ligand while 50 mM NaOH and 100 mM thioglycerol followed by 4 M guanidine hydrochloride reduced the concentration by just ~0.15 mg/mL after three cycles. Using 25 mM NaOH cycles showed no appreciable difference from the initial ligand density. Since the binding capacity should be proportional to the ligand density^36^, our approach could also be employed to predict the affinity to mAbs post-CIP without unpacking the column.

### In-column ATR-FTIR spectroscopy of null-cell fouling

Following depth filtration, Protein A affinity chromatography usually constitutes the first purification step in the capture of antibodies. In addition to the main constituent, the mAbs culture fluid contains amino acids from the feed media and host cell proteins (HCPs), DNA and other cell debris. To investigate the effect of the different constituents, we first analysed the culture fluid from non-mAb producing null-cell lines. The total protein concentration was estimated by BCA assay to 1.01 mg/mL.

**Figure 5:**
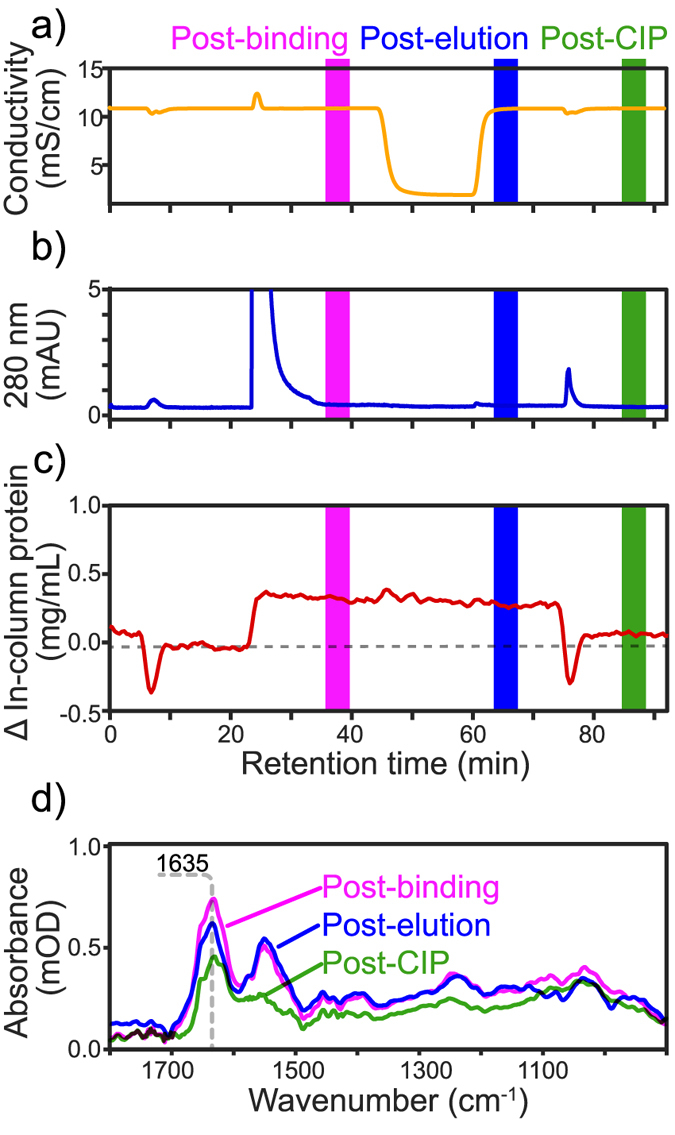
(**a**) Conductivity and (**b**) 280 nm absorbance of the mobile phase during purification of 500 μL of null-cell culture fluid and 50 mM NaOH CIP cycles. (**c**) In-column protein concentration calculated from ATR-FTIR spectra using PLS regression during purification and 50 mM NaOH CIP. (**d**) ATR-FTIR spectra after culture fluid binding, pH 3.0 elution and 50 mM NaOH CIP.

Figure 5a shows the conductivity measured on the outlet during the initial CIP, flow through, elution and final CIP cycle. After the strong flow through peak (610 mAU), no UV absorption was detected upon addition of pH 3.0 buffer (Fig. 5b). This result suggests that no protein bound to the resin or that they did not elute under low pH. The difference in protein concentration determined by ATR-FTIR spectroscopy answers this question in Fig. 5c (red curve). Following sample injection, the in-column protein concentration rose by ~0.4 mg/mL as some HCPs appear to bind to the Protein A resin. The concentration did not drop appreciably, during low pH elution suggesting strong affinity binding of the HCPs. Following CIP with 50 mM NaOH, most of the non-eluting protein is cleared from the column, but ~0.1 mg/mL of bound protein containments remained adsorbed on the resin. This result suggests that even harsher CIP conditions would be needed to completely remove all contaminant HCPs bound.

Figure 5d shows the difference in the ATR-FTIR spectra after each step, revealing the absorbance of the bound protein absorption. This contrasts with the spectra obtained for the whole null-cell culture fluid rich in amino acids shown in Fig. 2b. With a centre-of-gravity (COG) around 1631 cm^−1^, the amide I band position approaches that of pure mAbs suggesting a similar helical/unordered secondary structure. After CIP the amide I COG shifts to 1617 cm^−1^, suggesting that the remaining adsorbed proteins adopt a secondary structure with a higher β-sheet content, as previously reported for insoluble aggregates^16^,^49^,^50^. Only protein bands were observed, suggesting that binding contaminants were primarily proteins rather than lipids or DNA which should be clearly differentiated based on their unique IR bands.

**Figure 6:**
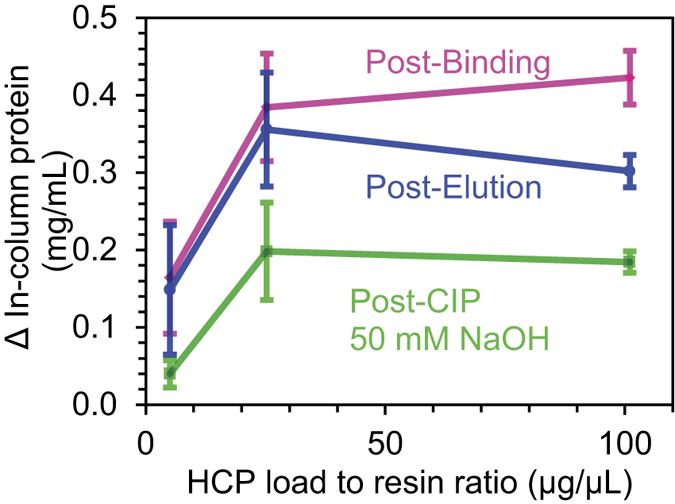
The difference in protein concentration determined by ATR-FTIR spectroscopy: post-binding (magenta), post-elution (blue) and post-CIP (green) as a function of HCP load. The error bars represent the 95% confidence interval for n = 3.

Figure 6 shows the difference in protein concentration in the column measured after binding, elution and CIP for three sample loadings. After loading an equivalent of 5 μg of HCP per μL of resin, the increase in protein concentration post-binding is barely detectable, but at 25 μg/μL the in-column protein concentration rose appreciably to ~0.4 mg/mL. Injecting 101 μg/μL of HCP did not appear to increase binding much further, suggesting that the amount might have exceeded the resin’s capacity to bind HCP contaminants. Low pH elution did not appear to decrease the in-column concentration much for all loads tested, confirming the strength of such interactions. CIP with 50 mM NaOH seems to have removed most HCP at 5 μg, but did not remove half of the adsorbed protein at either the 25 or 101 μg loads. These results, therefore, suggest that some HCP species strongly associate to the resin and remain adsorbed under typical CIP conditions. Such proteins could thus build-up over repeated cycles and restrict access to the Protein A ligand.

### *In situ* analysis of resin fouling

Previous reports suggest that full antibodies and mAbs fragments are also involved in Protein A resin fouling. After binding to the ligand, mAbs can bind HCPs via their antigen-binding Fab fragment or aggregate inside the porous matrix during elution^29^. To study the fouling process of Protein A affinity resin, our in-column ATR-FTIR spectroscopic setup measured mabSelect resin during purification and CIP of full culture fluid containing 0.75 mg/mL mAbs.

**Figure 7:**
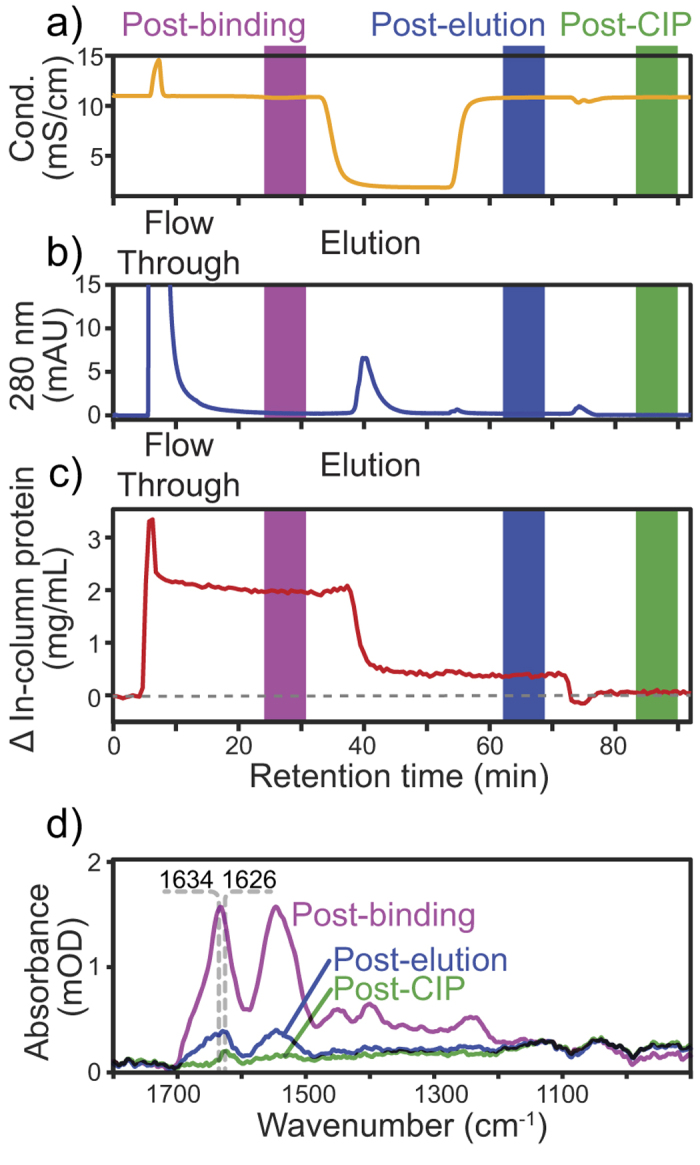
(**a**) Conductivity (orange) and (**b**) 280 nm absorbance (blue) of the mobile phase during purification of mAb culture fluid and 50 mM NaOH CIP cycles. (**c**) In-column protein concentration difference (red) calculated from ATR-FTIR spectra using PLS regression during purification and 50 mM NaOH CIP. (**d**) ATR-FTIR spectra after mAb binding, pH 3.0 elution and 50 mM NaOH CIP.

Following a first CIP cycle to condition the Protein A resin, Fig. 7a shows the conductivity measured during binding, elution and CIP. Because of the higher salt concentration in the culture fluid, injecting 500 μL of sample increased the conductivity before stabilising after the flow through peak. Compared to the binding buffer, the conductivity dropped upon addition of lower salt content elution buffer. The conductivity remained almost constant when 50 mM NaOH CIP buffer was added as the salt concentration was similar to the binding buffer.

Since mAbs bound to the Protein A resin, Fig. 7b shows a clear elution peak, reaching 6 mM under low pH buffer. When injecting 500 μL of culture fluid (38 mg of mAb per mL of resin), the area of the peak however represents only ~1% of the integrated 280 nm absorbance of the flow through, capturing only a small fraction of the mAbs product. The small column volume (10 μL) and high load could be responsible for the low capture ratio. Moreover at 0.4 mL/min, the residence time was only 1.25 minutes, at the lower end of typical values (1 to 6 min)^17^. The small geometry of the microcolumn would also make the wall effect greater^46^, increasing the linear velocity on the IRE surface. Cleaning the column with 50 mM NaOH resulted in a weak peak of merely 1 mA, indicating that some other proteins were removed through CIP.

As shown on Fig. 7c, the protein concentration quantified by ATR-FTIR spectroscopy complements UV 280 nm detection by revealing only what remains in the column after each step instead of what flows through. Probing the stationary phase had the benefit of allowing the subtraction of the bands from the amino acids in the culture fluid and thus measurement of only protein bound to the affinity resin. Following a 38 μg/μL (mAb/resin) culture fluid injection, the in-column protein concentration increased to ~2 mg/mL indicating that Protein A effectively bound to the Protein A resin. Application of the low pH buffer resulted in elution of most of the protein, but a sizable amount remained bound post-elution. These irreversibly bound proteins could be responsible for resin fouling and binding capacity decay. Applying 50 mM NaOH cleared most of the post-elution proteins demonstrating the efficiency of CIP.

Figure 7d shows the differences in the ATR-FTIR spectra after each step. As expected, the spectrum after binding resembles purified mAbs rather than culture fluid since the mAbs bound to the resin in the probed volume. After flowing buffer at pH 3.0 through, the in-column concentration dropped while the amide I band shifted to lower wavenumbers, suggesting that non-eluting proteins contained slightly more β-sheet than the mAb. After 50 mM NaOH CIP even less protein remains. With an even lower amide I band wavenumber, the difference spectra post-CIP indicates that the remaining fouling proteins are mainly comprised of β-sheet rich aggregates, in agreement with previous studies^16^,^68^.

**Figure 8:**
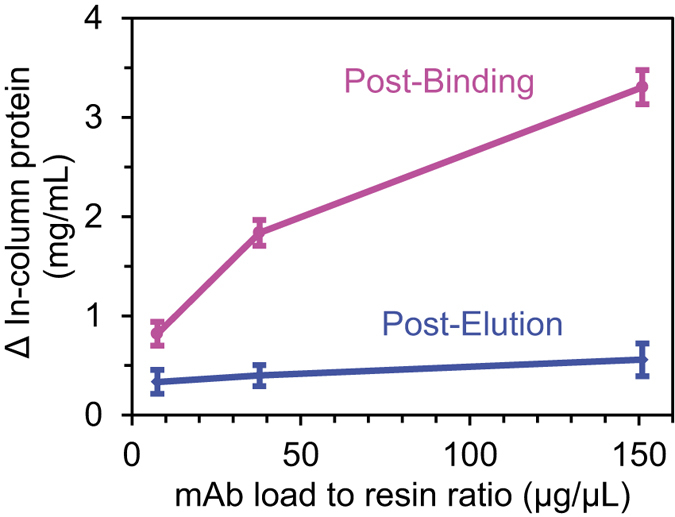
In-column protein concentration difference calculated from ATR-FTIR spectra using PLS regression post-binding (magenta), post-elution (blue) and post-CIP 50 mM NaOH as a function of mAbs load. The error bars represent the 95% confidence interval for n = 3.

Figure 8 shows the difference in protein concentration determined by ATR-FTIR spectroscopy post-binding and post-elution for three different mAb samples. As the mAb breaks through when the load exceeds the binding capacity (~30 mg/mL)^17^, the in-column protein concentration will plateau relative to the amount of mAb loaded. At 8 mg/mL of mAbs, the in-column protein concentration reaches 1 mg/mL after binding while loading 151 mg/mL only increased the in-column concentration to ~3.5 mg/mL. The proportion of irreversibly bound protein post-elution appears to decrease slightly with load from ~30 to 20%. Competition between mAbs and HCPs for the resin binding sites could explain this result. In addition to sodium hydroxide solutions, there are a range of different CIP protocols which have also proved effective at removing protein aggregates^28^,^69^.

**Figure 9:**
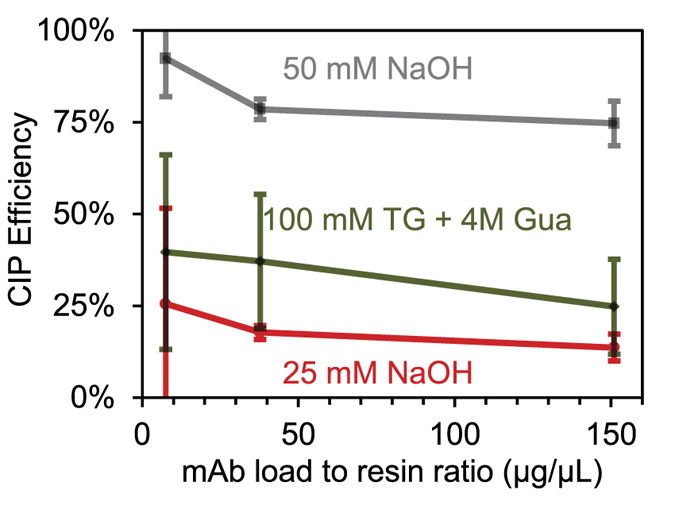
The efficiency of CIP at removing non-eluting protein as a function of mAb load. The error bars represent the two standard deviation interval for n = 3.

Figure 9a reveals the efficiency of three CIP procedures in removing bound protein which failed to elute at pH 3.0. At low load, 50 mM NaOH proved the most effective by removing almost all non-eluting proteins bound to the column while other protocols were less than 25% effective. Under greater load, the efficiency of 50 mM NaOH dropped to ~80% but remained much more effective than 25 mM NaOH and 100 mM thioglycerol (TG) followed by 4M guanidine hydrochloride (Gua). At a load much greater than the reported binding capacity (1510 μg), the efficacy of milder CIP buffers dropped to less than 10%. These results thus clearly reveal the inability of such conditions to remove contaminant proteins which may contribute to binding capacity decay.

## Discussion

Here we describe direct coupling of infrared spectroscopy with affinity chromatography for the first time, as a means of better understanding Protein A based affinity chromatography of mAbs. Our in-column ATR-FTIR spectroscopic setup allows direct analysis of the stationary phase during purification. This was complemented by the measurement of the mobile phase with UV 280 and conductivity using a standard protein purification system. The micro-scale column setup uses very little resin allowing us to very efficiently assess the effects of a number of different purification conditions and cleaning protocols. Since organic molecules absorb infrared light at specific frequencies, ATR-FTIR spectroscopy can differentiate between the different molecules within the column without the need for labelling. This allows differentiation between the agarose beads, protein, DNA and lipid in any sample. This approach provided an opportunity to characterise precisely what contaminants are fouling the resin and potentially leading to reduced binding capacity. When flowing either null-cell culture fluid from a non-antibody expressing cell line or cell culture fluid containing mAbs through the resin, ATR-FTIR detected contaminant protein binding to the resin. By measuring the stationary phase, our approach also revealed host cell proteins (HCPs) remaining after low pH elution for both samples. In the case of the null-cell culture fluid, over 0.5 mg/mL of HCP bound to the resin which did not elute at low pH.

Notably, larger quantities of contaminant bound when mAbs were present in the applied sample. A previous study by Zhang *et al*. hypothesised that mAb binding to the resin could undergo conformational change, forming a partially unfolded intermediate^29^. The mAb may then expose hydrophobic domains more likely to associate with lipophilic HCPs^29^. This association could then result in the formation of large HCP-mAb complexes prone to aggregating within the porous resin matrix. Infrared spectra collected after elution and CIP support this hypothesis by indicating that the remaining protein contains a larger proportion of β-sheets, known to form hydrophobic domains^36^,^50^. Identification of the individual contaminating species did not form part of this study. It is possible however that one of the contaminants is histone previously shown bind to protein A, reducing the dynamic binding capacity^8^.

Interestingly, our analysis did not detect any contaminant lipid or DNA. Thus future efforts to optimise the protein A purification process need to focus primarily on removal of contaminating proteins.

Our approach also allowed analysis of the effects of CIP protocols used to prevent resin fouling. By testing CIP alone, we were able to quantify the Protein A leaching from unused MabSelect SuRe resin over repeated cleaning cycles. The first exposure to CIP buffer leached much more ligand than subsequent cycles. While the underlying cause remains unclear, it is possible that the first CIP cycle ligand causes leaching of more weakly bound Protein A molecules. Hence, to avoid misinterpreting leaching protein A as cleared fouling contaminants in the mass balance calculation, we performed a CIP before exposing MabSelect resin to culture fluid.

We found that CIP cleared most of the contaminant protein although the efficiency was dependent on the CIP protocol used. However, even the most effective method tested, the standard 50 mM NaOH treatment, did not return the protein concentration to its initial value. Hence, there is scope for the development of more effective CIP protocols to reduce contaminant binding. Harsher CIP conditions are more effective but as we showed recently these reduce binding capacity by degrading the ligand^26^,^36^. Thus our in-column sensing approach could help optimization of the CIP protocol to achieve the best compromise between column cleaning and ligand degradation.

Our microchip setup demonstrated the usefulness of an in-column ATR-FTIR spectroscopic approach. It is important to note that the very small scale of the column could have accentuated the wall effect on the flow profile^46^,^48^. Further studies are required to confirm our findings in large scale set ups. However, it is anticipated that our approach could be easily be applied to full-scale chromatographic processes via the embedding of sensors in the column casing. Instead of using MCT detectors requiring liquid nitrogen cooling, the more common and affordable DTGS detectors could be employed to monitor purification processes. The angle of incidence of the infrared light in the diamond ATR accessory could be adjusted to optimise the penetration depth and the resin signal^65^,^70^. The addition of ATR-FTIR spectroscopic sensors to gel chromatographic columns offers many advantages to process analytical technology (PAT). Stationary phase composition monitoring could track the mass balance to predict binding capacity and optimize CIP protocols to individual feeds. Monitoring protein contaminant build-up with in-column PAT could also prove useful for quality assurance purposes. Beyond research and development, adjusting the CIP condition to the fouling state of the column could enhance resin lifespan and reduce resin replacement rate.

The application of non-invasive, label-free ATR-FTIR spectroscopy revealed that loss of binding capacity of the column is thus a combination of irreversible binding of host cell proteins with no detectable contribution to fouling from lipids or DNA. In addition, the approach has allowed a detailed comparison of the different CIP protocols. Even minor alterations in the purification and cleaning protocols could result in a significant cost saving in the production of therapeutic mAbs.

## Method

### Microchip for In-column ATR-FTIR spectroscopy

The microfluidic device was encased in a laser-cut PMMA sheet assembly with bolts. The microchip was made of a Sylgard^®^ 184 poly-(dimethylsiloxane) (PDMS) elastomeric substrate (Dow Corning Corporation, Midland, MI, USA). To prevent protein adsorption, the PDMS elastomer contained 1.5% % poly(dimethylsiloxane-ethylene oxide) before casting. The hydrophilic elastomer was cast from a PMMA 3D printed negative master made by multijet modeling with a layer resolution of 16 μm (Shapeways, New York, USA).

Figure 1a shows the microfluidic assembly containing the microchip with an inlet and outlet. A funnel was attached to the top port and buffer pumped through the cell to push the air out. The microchip was packed with 10 μL of sedimented MabSelect Sure resin (GE healthcare, Little Chalfont, UK) constituted of porous cross-linked agarose beads ranging from 20 to 160 μm in diameter. To ensure that the resin beads filled the entire column cavity, buffer was pumped through the top loading port to push the resin down.

Subsequently, the funnel was replaced by the plunger (Fig. 1b). The resin was slowly packed under 0.4 mL/min flow while collecting ATR-FTIR spectra until the absorbance of the main agarose band at 1060 cm^−1^ reached 50 mA. A 32 scan ATR-FTIR spectrum of the packed resin bed under 0.4 mL/min was collected as a reference background. The back pressure inside the device typically did not exceed 0.18 MPa under 0.4 mL/min flow. After completing the LC-IR *in situ* measurements, a new background single channel was collected before removing the plunger under flow. The unpacked resin beads flowed out of the column cavity before another 32 scan ATR-FTIR spectrum was collected.

The close-up view of Fig. 1c shows the nine channels with a width of 40 μm feeding the mobile phase into the microchip cavity of 1600 by 1200 by 300 μm constituting the micro-scale column. The base of the chromatographic column was probed by the evanescent wave generated from the internal reflection element (IRE) of the diamond attenuated total reflection (ATR) accessory (Specac, Orpington, UK). Diamond is an ideal IRE material as it is both resistant to corrosion and hard. A 45° angle of incidence resulted in a ~1.2 μm depth of penetration^65^,^70^. The walls of the microfluidic device were not within the probed volume as no peaks for PDMS were observed. ATR-FTIR pectra were collected using a Tensor 27 FTIR spectrometer (Bruker, Billeria, USA) equipped with globar silicon carbide infrared source, a KBr beam splitter and a liquid nitrogen cooled single-element MCT detector. Data were collected with a spectral resolution of 4 cm^−1^. Spectra were the result of co-adding 32 scans that required 37 seconds.

The mobile phase was pumped through the microfluidic device at 0.4 mL/min using an Akta Prime liquid chromatography instrument operated by PrimeView (GE Healthcare, Little Chalfont, UK). The 10 mM phosphate binding buffer was adjusted to pH 7.4 and contained 100 mM NaCl. The pH 3.0 elution buffer did not have added NaCl to reduce binding affinity to mAbs. Sodium hydroxide solutions of 25–400 mM were used for CIP. To replace depth filtration typically used for large scale purification^2^,^11^, the culture fluid samples were filtered through 0.4 μm syringe filter before injection to remove large particulates.

### mAb sample preparation

The chimeric B72.3 immunoglobulin G gamma 4 (IgG4) was expressed in a Glutamine Synthetase Chinese Hamster Ovary (GS-CHO) cell line (Lonza Biologics, Basel, Switzerland). Cultures were maintained in protein-free, serum-free chemically defined CD-CHO medium (Invitrogen, UK) with 25 mM L-Methionine sulfoximine (Sigma, UK) at 36.5 °C with 8% CO_2_ air while stirred on an orbital shaker at 140 rpm. In batch cultures conducted in 1 L Erlenmeyer flasks with a working volume of 300 mL, cells were subcultured every 3 or 4 days to achieve a target seeding density of 2 × 10^5^ cells/mL. The mAb concentration in the media samples was quantified by ELISA (Montgomery, TX, US) and UV spectrophotometry using a Nanodrop Lite system (Thermo, Wilmington, DE, USA) and a E^1%^ of 13.7. Samples were then stored at −80 °C until further use.

The mAb standards were defrosted and filtered through a 0.45 μm filter disk to remove large particulates. Subsequently, the culture fluid containing the product protein and host cell proteins was either used directly in the set-up described above or the mAb was purified using affinity chromatography with a MabSelect SuRe Protein A column (GE Life Sciences, UK). The concentration of mAb standard solutions were quantified by UV absorption at 280 nm with a Nano drop Lite (Thermo, USA) using E^1%^ of 13.7, corresponding to 260.4 mA/mL on the FPLC chromatograms. The 0.75 mg/mL mAb concentration was calculated from the eluted fraction after Protein A affinity chromatography. The total protein content in the culture fluid was quantified using the bicinchoninic acid (BCA) assay by measuring the 562 nm absorbance compared to BSA standard solutions. The null-cell culture fluid had a total protein concentration of 1.02 mg/mL while the mAb culture fluid had a concentration of 0.75 mg/mL.

### Resin fouling measured by in-column ATR-FTIR spectroscopy

A background spectrum was collected during equilibration to subtract the absorbance from the hydrated resin bed. ATR-FTIR spectra were collected continuously during the purification and cleaning-in-place cycles. The LC-IR purification of IgG, 500 μL of raw culture fluid sample was pumped from the injection loop before washing unbound IgG with 10 mL (1000 CVs) of binding buffer. Bound mAb was eluted with pH 3.0. For CIP cycles, 200 μL of 25, 50 or 400 mM NaOH or 100 mM thioglycerol followed by 4 M guanidine hydrochloride were flowed through the column prior to equilibration with 10 mL of binding buffer.

### Data analysis

ATR-FTIR spectra were exported from Opus (Bruker, Billeria, USA) and chromatograms exported from Unicorn (GE healthcare, Little Chalfont, UK) to MATLAB (MatWorks, Natick, MA, USA). Using a custom made MATLAB code, spectra were offset using the average absorbance values between 1870 and 1840 cm^−1^. Spectra kinetics were averaged using a mobile window of 4 spectra of 32 scans. A 5-point smoothing function was applied on the spectra presented on Fig. 5. The adsorbed protein concentration and resin pH were quantified using a partial least square (PLS) regression based on concentration standards. Spectra of purified IgG solutions of 0.5, 1, 2, 4 and 8 mg/mL were used for the adsorbed protein concentration curve. The partial least square (PLS) method used the 1800 to 1400 cm^−1^ region to quantify adsorbed protein concentration (PLS model R^2^ = 0.89). Finally, the in-column ATR-FTIR spectroscopic data, conductivity and UV 280 nm data were coupled in a database using time as common variable.

## Additional Information

**How to cite this article**: Boulet-Audet, M. *et al*. In-column ATR-FTIR spectroscopy to monitor affinity chromatography purification of monoclonal antibodies. *Sci. Rep*. **6**, 30526; doi: 10.1038/srep30526 (2016).
